# Pulmonary Loiasis and HIV Coinfection in Rural Cameroon

**DOI:** 10.1371/journal.pntd.0000572

**Published:** 2010-03-30

**Authors:** Alexis Cambanis

**Affiliations:** 1 St. Elizabeth Hospital, Shisong, Cameroon; 2 Tropical Medicine Program, PIH-UCI Family Medicine Residency, Whittier, California, United States of America; Emory University, United States of America

## Presentation of Case

A 38-year-old man presented to a rural hospital in Northwest Cameroon with a one-month history of dyspnea that worsened upon exertion. He reported occasional cough, which was nonproductive. Review of systems revealed a fall in weight from 81 kg to 71 kg over the last year, but no other problems were noted. The patient initially sought care at a health center in Douala (10 hours away by public transport), where he was treated for typhoid with no improvement.

**Figure 1:**
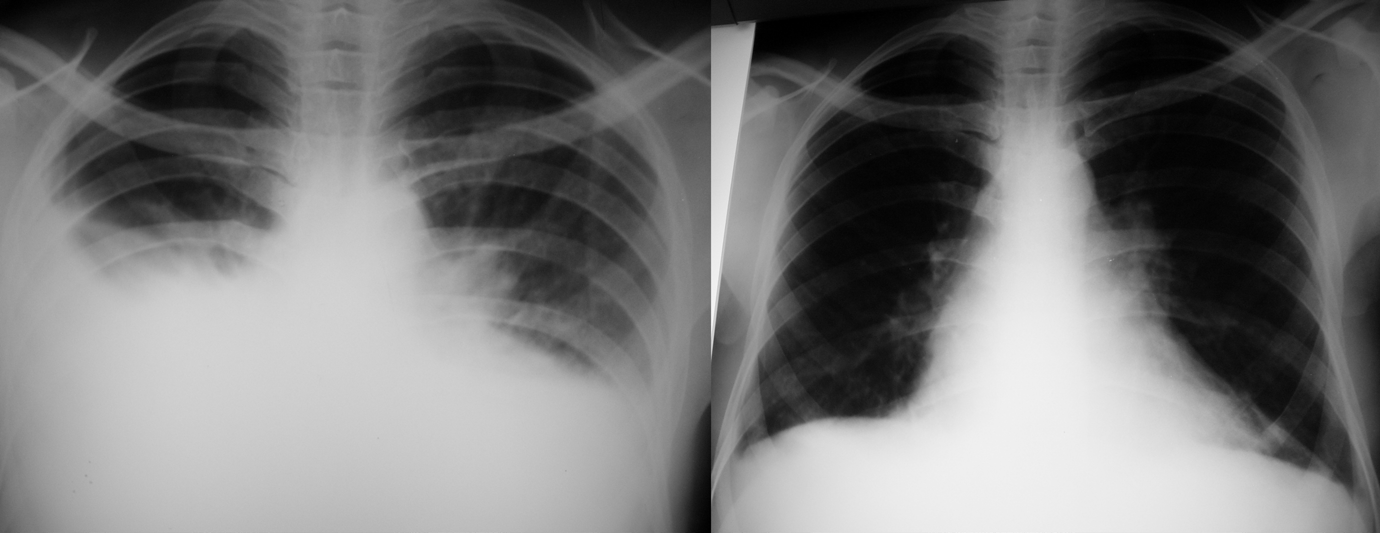
Resolution of pleural effusions after treatment for loiasis. Chest radiographs showing bilateral basilar densities at the time of admission (left) and complete resolution at 3-week follow-up after one dose of ivermectin (right).

**Figure 2:**
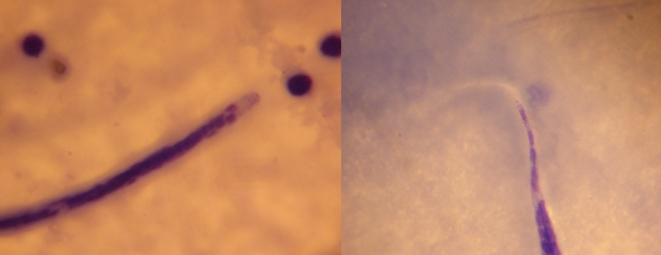
*Loa loa* found in pleural aspirate of HIV-positive man in Cameroon. Microfilaria of *Loa loa* seen in Giemsa-stained preparation of pleural fluid showing morphological characteristics including sheath and terminal nuclei (×1,000). Note that Giemsa does not specifically stain the sheath, but its outline is clearly visible in this preparation. Several lymphocytes can also be seen.

On physical examination, temperature was 37.8°C, blood pressure 110/70 mmHg, pulse 88 beats per minute, and respiratory rate 28 breaths per minute. He was noncachectic without thrush or lymphadenopathy. Lung auscultation revealed no bronchial breath sounds or wheezes, but decreased breath sounds and dullness to percussion were noted at both lung bases, and a chest radiograph showed dense bibasilar opacities (Figure 1). Diagnostic thoracentesis confirmed the presence of exudative pleural effusion; cytological examination yielded 435 white blood cells/mm^3^ (84% lymphocytes, 10% neutrophils, and 6% eosinophils), 240 red blood cells/mm^3^, and numerous motile microfilariae (mff) throughout the specimen (see Video S1). A smear was made of the pleural aspirate and the filarial species identified as *Loa loa* based on morphological features (Figure 2). Other laboratory investigations included hemoglobin of 9.3 mg/dl, white blood cells 9,800/mm^3^, a thick blood film negative for malaria parasites, and a positive HIV serology test. His CD4 count was 954 cells/µl. Peripheral blood was not examined for microfilariae.

After discussing these results with his physician, the patient received a single dose of ivermectin (150 µg/kg body weight) and all other medications that had been ordered by the admitting team—including multiple broad-spectrum antibiotics, ventolin, and amodiaquine—were stopped. A short course of prednisone was initiated to reduce potential reaction to parasite antigens. The next day, he left the hospital in stable condition. Follow-up examination three weeks later showed normalization of his vital signs and total resolution of the pleural effusions on repeat radiography (Figure 1). The patient was encouraged to participate in the annual ivermectin campaigns common in the area and to monitor his HIV infection at his local medical center.

## Case Discussion

### Loiasis in sub-Saharan Africa

*Loa loa* is a filarial nematode transmitted by *Chrysops* tabanid flies and is limited to West and Central Africa. Most infected people remain asymptomatic, but after a latency period from 6 months to several years, clinical symptoms may develop, most often intermittent edematous lesions in the extremities (Calabar swellings) or passage of the adult worm under the conjunctiva [1]. It is estimated that between 2 and 13 million people are infected with *Loa loa*, and a recent survey in the Eastern Province of Cameroon showed a microfilarial prevalence of over 20% in most villages and over 45% in several areas, with twice that level of clinical manifestations [2]. There have been rare reports of pulmonary involvement manifested as eosinophilic infiltrates [3], [4] in Cameroon and one case of pleural effusion that contained microfilariae of *Loa loa* in a Ghanaian patient who had traveled extensively in West Africa [5], but this case is the first to describe pulmonary loiasis in a known HIV-positive patient.

### Treatment options

Effective treatment of loiasis includes diethylcarbamazine (DEC) administered over 2–4 weeks or a single dose of ivermectin, but DEC is the only drug with both micro- and macrofilaricidal activity [1]. Definitive cure sometimes requires repeated courses of DEC as some adult worms survive the first treatment, and side effects such as itching, rash, headache, and fever are common. The microfilaricidal effect of ivermectin lasts for over a year. Both treatments can cause an often fatal encephalopathy, especially in people with high (30–50,000 mff/ml) microfilaremias, characterized by aphasia, extrapyramidal signs, incontinence, and retinal hemorrhage. In heavily infected patients, the mff load can be reduced by a 3-week course of albendazole, three sessions of apheresis, or ivermectin prior to administering curative DEC more safely [1].

When used for onchocerciasis control in Cameroon, ivermectin caused functional impairment in ∼0.1% of cases and serious neurological events in 1 per 10,000 patients, and it is estimated that microfilaremias above 8,000 mff/ml confer a risk of postivermectin impairment [6]. This patient was counseled about available therapies as well as their risks, and he chose ivermectin so as not to remain in the hospital or on prolonged drugs. *Loa loa* does not harbor symbiotic *Wolbachia* like some nematodes, so doxycycline plays no role in treatment of loiasis [7].

### Helminth–HIV coinfection

The high prevalence of loiasis in Cameroon mentioned above coexists with an HIV prevalence estimated at 5.1% in 2007 [8]. The hypothesis that helminth infections not only adversely affect the progression of HIV disease but also increase susceptibility to HIV infection in the first place is garnering increasing attention [9]. Normal immune responses to HIV may be hampered by chronic immune activation (of the T helper cell 2 type) and anergy. Indeed, peripheral blood mononuclear cells from people with filarial infections are more susceptible to HIV infection in vitro [10]. Several prospective cohort studies suggest helminth eradication in HIV-positive patients is associated with reduced viral loads [11], and a recent randomized control trial in Tanzania showed significant beneficial effect on HIV viral load after a course of DEC [12].

## The Presenting Case

The case described here illustrates several important points for clinicians in the tropics. The initial management of the patient was not as tailored as it could have been, with intravenous antibiotics, antimalarials, and beta-agonists given all at once in a kind of “shotgun” approach. While empiric treatment for both malaria and pneumonia is common in such settings, admitting nurses and attending doctors often rely upon algorithmic treatment protocols not necessarily based on clinical data. In this case, accurate initial physical exam findings would have steered the diagnosis in a more specific direction if the pleural fluid had been promptly analyzed.

In sub-Saharan Africa, most cases of lymphocytic hemorrhagic exudative pleural effusion are tubercular, and, pending pleural fluid analysis, the ward physician was planning to initiate therapy for extrapulmonary tuberculosis (TB). Differential diagnosis of such effusions includes mainly malignancy—from primary bronchogenic to metastatic processes—and, more rarely, fungal or resolving bacterial infection. HIV-positive patients who worsen on TB treatment with ongoing hemorrhage often have pulmonary Kaposi's sarcoma. The unexpected finding of *Loa loa* microfilariae in this effusion reminds us that other explanations are possible, especially in HIV-positive patients who do not improve with initial therapy or whose CD4 count implies a relatively recent seroconversion. Most often the causes of exudative lymphocytic effusions discussed above produce unilateral disease, and this patient had bilateral findings, which may be an indicator of atypical etiology. Another possibility is that pleural microfilariae are incidental findings in endemic areas, but the patient's recovery without other treatment regimes supports a single diagnosis, and antifilarial therapy led to rapid clinical improvement in several prior cases of pulmonary loiasis [3], [4].

Pulmonary manifestations of *Loa loa* infection include pulmonary infiltrates and eosinophilic pleural effusion [1]. Once the diagnosis was established, the physician suggested a course of DEC, but the patient no longer wished to remain in the hospital, despite being made aware of potential severe adverse drug reactions such as encephalitis. He agreed to stay for 24 hours after receiving the ivermectin for monitoring as a compromise.

Although loiasis is the second or third most common cause for consultation in parts of Central Africa, it has received relatively less attention than even other filarial neglected tropical diseases [1]. The case presented here serves as a reminder of the high prevalence of *Loa loa* in Cameroon and that it can generate serious yet atypical clinical manifestations. In this HIV–*Loa loa* coinfected patient, it is impossible to ascertain which infection came first, but the high CD4 count and early clinical stage suggests that the HIV infection was relatively recent. Concerns raised by the literature regarding immune activation during helminth infection and the resulting possibility that people infected with helminths are more susceptible to HIV infection make it that much more imperative for clinicians in the tropics to be vigilant in their search for accurate diagnoses, to be aware of locally prevalent diseases and their treatment, and to say “yes” to the question “should we deworm?” [9] when these helminths are encountered.

**Learning Points**

- Not all lymphocytic pleural effusions in sub-Saharan Africa are due to tuberculosis or malignancy.
- Eosinophilic effusions must be examined for microfilariae, and high CD4 counts in HIV-positive patients with pleural effusion mandate broader differential diagnosis and evaluation in areas where filariae are prevalent.
- There is increasing attention in the literature to the interaction between HIV and helminths, and recent studies suggest helminth eradication may reduce plasma viral load in coinfected patients.
- Caution must be exercised when using ivermectin in areas endemic for *Loa loa*, as severe reactions including encephalopathy have been reported in patients with very high microfilaria loads.
- Although more geographically restricted than other filarial diseases, loiasis remains highly prevalent in parts of Central Africa and is the second or third most common cause of medical consultation in some areas.

## Supporting Information

Video S1 Live *Loa loa* in fresh pleural fluid. Motile microfilaria seen in fresh preparation of pleural aspirate seen under low power prompted further investigation of disease etiology.
(5.59 MB AVI)
